# Identification and Expression Analysis of Rice MYB Family Members in Response to Heat Stress

**DOI:** 10.3390/plants14121784

**Published:** 2025-06-11

**Authors:** He Zhao, Yaliang Ji, Yaohuang Jiang, Xiao Liang, Yu Qiao, Fei Chen, Limin Wu, Yanchun Yu, Dianrong Ma

**Affiliations:** 1 Rice Research Institute, Shenyang Agricultural University, Shenyang 110866, China; 2 Beijing Compass Biotechnology Co., Ltd., Beijing 102209, China; 3 College of Life and Environmental Sciences, Hangzhou Normal University, Hangzhou 311121, China; 4 Agronomy College, Liaodong University, Dandong 118003, China; 5 Liaoning Academy of Agricultural Sciences, Shenyang 110161, China

**Keywords:** rice (*Oryza sativa* L.), MYB transcription factor, heat stress

## Abstract

With the continuous rise in global temperatures, heat stress has become a significant threat to rice (*Oryza sativa* L.) growth and yield. MYB transcription factors, the largest family of genes in plants, play a crucial role in mediating responses to various abiotic stresses. However, the specific functions of MYB genes in rice under heat stress remain largely unexplored. In this study, we conducted a comprehensive genome-wide characterization of the MYB transcription factor family and performed an RNA-seq analysis to identify OsMYB genes that are responsive to heat stress. We identified 229 MYB genes in rice, 134 of which exhibited significant expression changes under heat treatment. An RT-qPCR analysis validated the RNA-seq results for 15 MYB genes, confirming significant expression changes, such as the upregulation of *Os02g0685200* after heat stress and the downregulation of *Os05g0579600*. Six highly responsive genes were selected for further analysis. *Cis*-acting elements associated with hormone response and abiotic stress were identified in the promoter regions of these genes. A subcellular localization analysis revealed that, except for *Os05g0579600*, which located to both the nucleus and cytoplasm, the other MYB genes (*Os01g0192300*, *Os02g0685200*, *Os06g0637500*, *Os06g0669700,* and *Os09g0106700*) were predominantly located in the nucleus. In yeast, *Os01g0192300*, *Os06g0637500*, and *Os06g0669700* exhibited transcriptional activation activity, while *Os02g0685200* and *Os09g0106700* showed transcriptional repression activity. Notably, these genes responded not only to heat stress but also to other abiotic stresses, such as cold, salt, and heavy metal cadmium. This study provides valuable insights into the functional roles of OsMYB family genes in the heat stress response, identifying *Os01g0192300*, *Os02g0685200*, *Os05g0579600*, *Os06g0637500*, *Os06g0669700*, and *Os09g0106700* as potential key genes involved in heat tolerance in rice.

## 1. Introduction

According to the World Meteorological Organization (WMO), each year since 2015 has set a new record for the highest global temperatures ever recorded [1]. Specifically, in 2024, the global monthly average temperature remained 1.55 °C above pre-industrial levels for most of the year [1]. The continuous rise in temperature significantly impacts agricultural productivity, particularly for heat-sensitive crops like rice. Heat stress inhibits photosynthesis, root development, and leaf expansion during the vegetative stage of rice, while it impairs pollen viability, fertilization, and grain filling during the reproductive stage—ultimately leading to significant yield and quality losses [2,3,4,5,6]. Consequently, improving heat tolerance in crops has become increasingly vital [7,8].

In response to heat stress, plants activate a complex regulatory network, with transcription factors (TFs) playing a pivotal role in triggering the expression of downstream heat-resistant genes. Heat Shock Factors (HSFs) are key regulatory factors that respond to heat stress in plants [9,10]. For instance, over expression of *HSFA1* significantly enhances heat tolerance, while knockouts of this gene exhibit increased sensitivity to high temperatures [11]. In crops, numerous HSF genes contribute to improved heat tolerance in transgenic plants [12,13,14,15,16].In addition to HSFs, other transcription factor family also play a crucial role in the plant response to heat stress. *OsNAC8* (*OsNTL3*) is vital for heat stress tolerance in rice seedlings, transmitting signals from the endoplasmic reticulum and plasma membrane to the nucleus to regulate stress-responsive genes [17]. *ZmNAC074*, a homolog of *OsNAC8* in maize, enhances heat tolerance by upregulating ROS-scavenging, heat shock, and UPR genes in transgenic Arabidopsis [18]. *ONAC127* and *ONAC129* can function both independently and synergistically by forming heterodimers to regulate rice grain filling and the heat stress response [19]. *OsWRKY11* is induced by heat treatment, and transgenic seedlings demonstrate significant heat tolerance [20].

The MYB transcription factor family is crucial for plant responses to abiotic stresses [21,22,23]. In *A. thaliana*, heat-related studies that focus on MYB involvement in heat stress responses have revealed several distinct functions. The *atmyb68* mutant retains a high seed-set rate during flowering under heat stress, whereas *AtMYB68* over-expression further enhances heat tolerance but simultaneously increases abscisic acid (ABA) sensitivity [24]. AtMYBS1 functions as a negative regulator of heat tolerance in Arabidopsis by suppressing the biosynthesis of strigolactones (SLs) through the direct inhibition of *MAX1* gene expression, thereby disrupting the SL signaling pathway, which in turn affects the plant’s ability to tolerate heat stress [25]. MYB30 directly regulates *ANN1* and *ANN4*, repressing their expression and reducing the calcium signaling mediated by these genes under oxidative and heat stress [26]. Consequently, *myb30* mutants exhibit increased sensitivity to oxidative stress but enhanced heat tolerance under heat stress [26]. In rice, *OsMYB55* (also termed *OsPL*) is one of the best-characterized heat-responsive MYB members. By binding the promoters of *OsGS1;2* (glutamine synthetase), *GAT1* (glutamine amidotransferase), and *GAD3* (glutamate decarboxylase), OsMYB55 upregulates their transcription, expands total amino acid pools, and enhances seedling thermotolerance through elevated metabolic fluxes [27]. Heterologous expression of *OsMYB55* in maize confers similar heat tolerance [28]. Strikingly, a germination-stage investigation reached the opposite conclusion: loss-of-function *osmyb55* mutants exhibited stronger thermotolerance during germination, in sharp contrast to the reduced tolerance observed in over-expression lines at the seedling stage [29]. These contrasting findings indicate that the role of OsMYB55 in thermotolerance requires further investigation because different developmental stages produced opposite phenotypes. A recent study identified the MYB61–UGT706F1 module, which promotes rice thermotolerance by coordinating flavonoid glycoside accumulation and activating heat-responsive genes (HSFs, HSPs, GSTs, and antioxidant enzymes), however, direct genetic evidence linking *OsMYB61* to heat stress remains unavailable [30]. While gene response patterns have revealed the dynamic regulation of MYB genes under heat stress (e.g., upregulation of *OsMYB30/OsMYB38* and suppression of *MYBS2* [31,32,33]), a critical gap persists in connecting these expression patterns to functional thermotolerance mechanisms, as neither gain- nor loss-of-function assays have substantiated their causal roles in heat adaptation.

Despite previous studies demonstrating the importance of MYB transcription factors in heat tolerance in *A. thaliana* and rice, the MYB family members involved in the heat stress response in rice have not been thoroughly explored. In this study, the rice variety “Zhonghua 11” was used for an RNA-seq analysis to examine gene expression changes in rice seedlings under heat stress. This study aims to identify novel members of the MYB transcription factors involved in the response to heat stress in rice seedlings, and will provide a theoretical basis for breeding heat-tolerant rice varieties and a basis for further improving rice heat tolerance.

## 2. Results

### 2.1. Identification and Phylogenetic Analysis of MYB Transcription Factors

Through a comprehensive analysis using BLASTp and HMMsearch (Hidden Markov Model search) methods, a total of 229 MYB transcription factors containing MYB domains (PF00249) were identified in rice (Table S1). For the phylogenetic analysis, we retained only the longest transcript for each MYB gene to construct a phylogenetic tree (Figure S1).

### 2.2. Expression Patterns of OsMYB Genes in Response to Heat Revealed by Transcriptome Profiling

**Figure 1:**
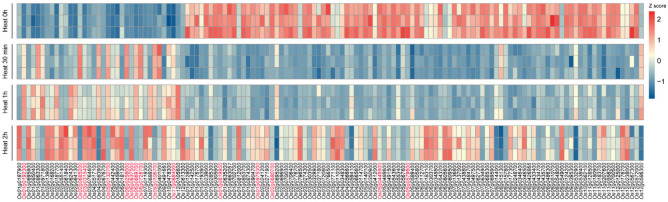
Heatmap of significantly differentially expressed MYB transcription factors after heat treatment. The heatmap illustrates the transcriptional changes of MYB transcription factors following heat treatment. The color gradient represents changes in gene expression levels, with red indicating upregulated expression and blue indicating downregulated expression. Three biological replicates were performed for each treatment time point to ensure the reliability and reproducibility of the results. All replicate FPKM values at the four time points are provided in Supplementary Table S2.

The RNA-seq data analysis revealed that among the 229 identified MYB family members, 134 genes exhibited significant expression changes following heat treatment (45 °C, 80% humidity). These genes were categorized into two major groups based on their expression patterns: upregulated and downregulated (Figure 1). The upregulated genes were further classified into three distinct expression patterns:

- Genes showing a rapid induction within 30 min to 1 h of heat stress, followed by a decline after 2 h (e.g., *Os01g0971800* and *Os06g0258000*);
- Genes displaying a delayed response, with peak expression occurring after 2 h of treatment (e.g., *Os01g0695900* and *Os02g0618400*);
- Genes exhibiting continuous upregulation throughout the 2 h heat stress period (e.g., *Os01g0192300* and *Os11g0684000*).

The downregulated MYB members were divided into two types:

- Genes showing an immediate downregulation followed by a gradual recovery to baseline levels (e.g., *Os02g0648300* and *Os03g0720800*);
- Genes maintaining sustained suppression throughout the heat stress period without recovery (e.g., *Os02g0680700*, *Os05g0449900*, *Os05g0579600* and *Os12g0572000*).

Notably, the second type of downregulated genes constituted a substantial proportion of all differentially expressed genes. The complete gene expression levels and differential analysis results are presented in Table S2.

### 2.3. Validation of OsMYB Gene Expression Profiles Under Heat Stress

**Figure 2:**
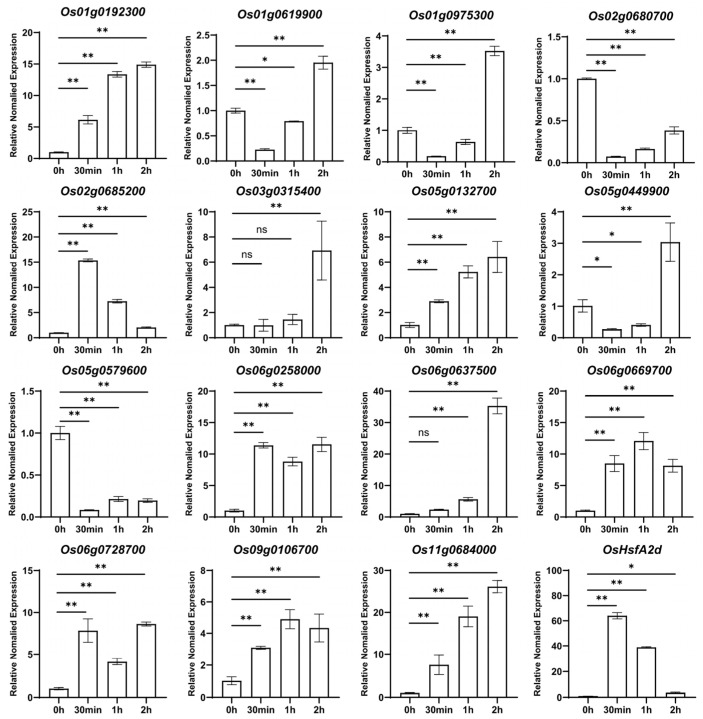
The expression pattern of OsMYB genes after heat treatment for 2 h in rice. The figure illustrates the RT-PCR analysis results for 15 OsMYB transcription factors under heat treatment conditions. The height of the bars indicates the relative expression levels, with the expression at the 0 time point set to 1. The asterisks indicate significant differences (* for *p*-value < 0.05, ** for *p*-value < 0.01), and ns indicates no significant difference. *OsHsfA2d* was used as marker gene.

To validate RNA-Seq findings, we performed an RT-qPCR analysis on 15 genes showing significant expression changes under heat stress conditions (Figure 2). The combined RNA-Seq and RT-qPCR results confirmed that heat treatment significantly affected the expression of seven genes: *Os02g0680700*, *Os02g0685200*, *Os03g0315400*, *Os05g0579600*, *Os06g0637500*, *Os06g0669700*, and *Os09g0106700*. Three genes (*Os01g0192300*, *Os05g0132700*, and *Os11g0684000*) showed significant upregulation within 30 min to 1 h of heat treatment, consistent with the RNA-Seq results. Conversely, *Os01g0975300*, and *Os05g0449900* exhibited significant downregulation during the initial heat stress period (30 min to 1 h), aligning with the RNA-Seq data. However, these genes showed an unexpected upregulation after 2 h of heat treatment, suggesting potential time-specific expression patterns that warrant further investigation. Two genes (*Os06g0258000* and *Os06g0728700*) exhibited irregular expression fluctuations that were inconsistent with the RNA-Seq results, and were consequently excluded from further analysis in this study. The observed discrepancies between the RNA-Seq and RT-qPCR results for some genes highlight the importance of complementary validation approaches in gene expression studies.

### 2.4. Characterization of Physicochemical Properties and Cis-Acting Regulatory Elements in Heat-Responsive OsMYB Genes

After rigorous validation of their heat-responsive transcriptional dynamics, we prioritized six candidate genes for subsequent functional characterization and mechanistic investigation; each displays pronounced transcriptional shifts under heat stress and has either not been studied before or has only been examined in contexts unrelated to heat stress, thereby ensuring both biological relevance and novelty. The physicochemical properties of these genes, including their gene loci, nucleotide lengths, peptide chain lengths, predicted molecular weights, and isoelectric points, are summarized in Table 1. The encoded proteins of these six OsMYB genes exhibit molecular weights ranging from 10.3 to 51.6 kDa, with protein lengths varying from 91 to 492 amino acids. The predicted isoelectric points (pI) span a range from 6.51 to 10.21, indicating diverse biochemical properties among these transcription factors.

**Figure 3:**
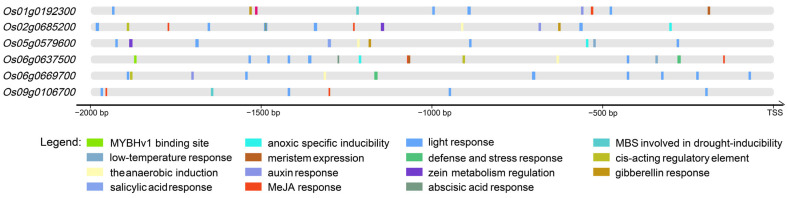
The analysis of the *cis*-acting elements in the promoter regions of OsMYB genes. The figure illustrates the major *cis*-acting elements identified in the promoter regions of six OsMYB genes. The different types of *cis*-acting elements are represented by distinct colors.

*Cis*-acting elements serve as crucial regulatory components governing stress responses and tissue-specific gene expression in plants. In this study, we conducted a comprehensive analysis of genomic sequences spanning 2000 bp upstream of the start codons, identifying three principal categories of *cis*-acting elements (Figure 3, Table S3):

- Abiotic stress response elements;
- Plant hormone response elements;
- Growth/development regulatory elements.

Our findings particularly emphasize the involvement of these genes in response to abiotic stress. Specifically, we identified low-temperature response elements in the promoter regions of *Os05g0579600*, *Os06g0637500,* and *Os06g0669700*. Furthermore, drought-inducible elements (MBS elements) were detected in the promoter regions of *Os01g0192300*, *Os06g0637500,* and *Os09g0106700*. Notably, all six investigated genes were found to contain anaerobic induction elements within their promoter regions, suggesting a conserved regulatory mechanism for a hypoxia response among these MYB transcription factors.

### 2.5. Hormone Treatment Expression Profiles of Heat-Responsive OsMYB Genes

**Figure 4:**
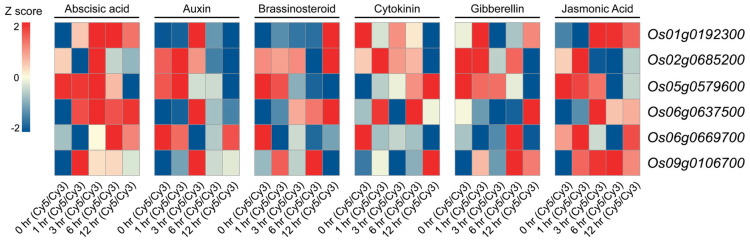
The heatmap of OsMYB gene expression in the stem under different hormone treatments. The color gradient represents changes in gene expression levels, with red indicating upregulation and blue representing downregulation. Cy5/Cy3 refers to the fluorescence intensity ratio obtained from the two-color microarray platform on which the RicExPro dataset is based.

To further elucidate the functional characteristics of the identified genes, we systematically analyzed their expression profiles under various plant hormone treatments (these hormone-response profiles were extracted from the public RicExPro database rather than generated de novo in this study). As shown in Figure 4, *Os01g0192300* exhibited strong induction following abscisic acid (ABA) treatment, which correlates with the presence of an ABA-responsive, *cis*-acting element located 64 bp upstream of its transcription start site (TSS). *Os02g0685200* demonstrated significant downregulation at 12 h post-cytokinin treatment. Notably, *Os05g0579600* showed contrasting responses to different hormones, being significantly induced by cytokinin treatment, while strongly suppressed by ABA. *Os06g0637500* was markedly upregulated in response to auxin treatment, and *Os09g0106700* displayed strong activation under both ABA and jasmonic acid treatments. These findings highlight the intricate regulation of OsMYB genes by various hormones, underscoring their potential roles in plant stress responses.

### 2.6. Subcellular Localization of Heat-Responsive OsMYB Genes

**Figure 5:**
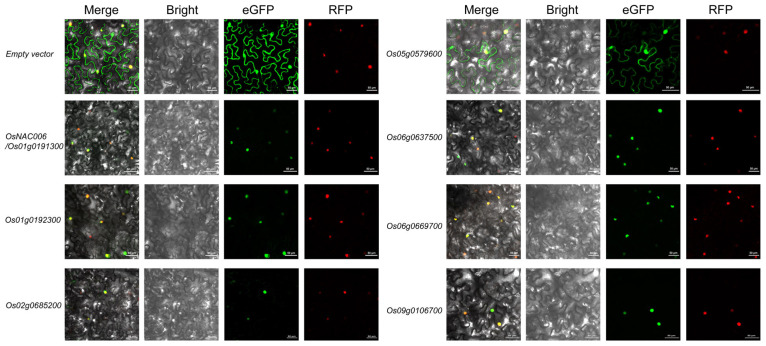
Subcellular localization analysis of MYB transcription factor family members. This figure illustrates the localization of each MYB gene and the empty vector (carrying eGFP). It includes images from the Merge, Bright, eGFP, and RFP channels. eGFP is used to mark the localization of MYB proteins, while RFP labels the nuclear localization signal. The scale bar is located in the bottom-right corner of each subfigure. The known nuclear localization of NAC006-GFP was used as a positive control.

To determine the subcellular localization patterns of these genes, we constructed plasmid vectors encoding fusion proteins by cloning their coding sequences with eGFP reporter gene. An empty GFP vector served as the negative control, showing diffuse fluorescence throughout the cell, including the nucleus. Nuclear localization was confirmed using mCherry as a nuclear marker, which emitted exclusive red fluorescence in the nucleus. The Os01g0192300-GFP fusion protein exhibited predominant nuclear localization, consistent with typical transcription factor behavior. Similarly, Os02g0685200, Os06g0637500, Os06g0669700, and Os09g0106700 primarily displayed nuclear localization (Figure 5). Interestingly, Os05g0579600-GFP showed dual localization in both the nucleus and cytoplasm (Figure 5), suggesting potential roles beyond conventional transcriptional regulation. These findings indicate that while most studied OsMYB genes exhibit canonical nuclear localization, *Os05g0579600* may have additional cytoplasmic functions.

### 2.7. Transcriptional Activity Using Yeast System

**Figure 6:**
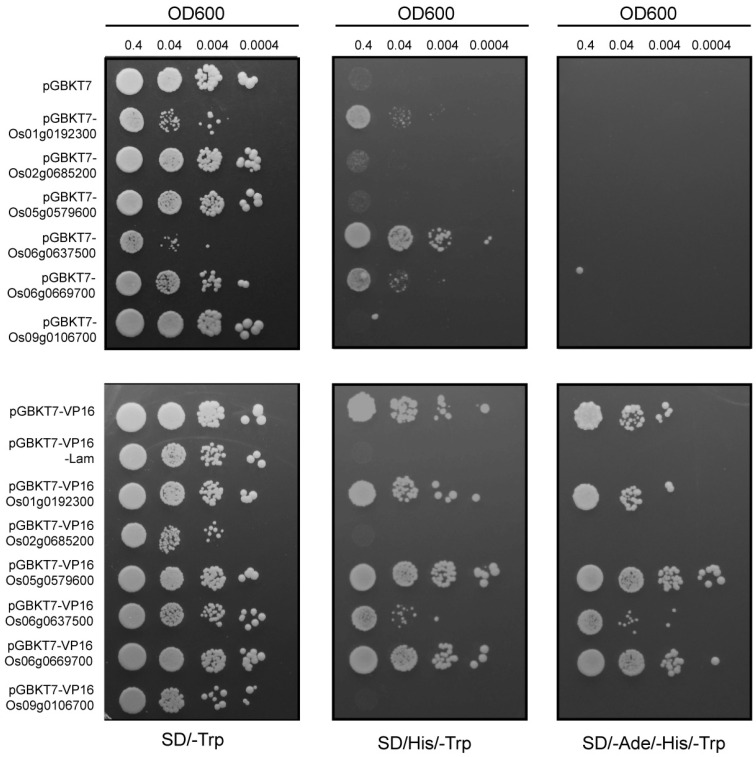
Transactivation analysis of OsMYB using yeast assay. Yeast clones containing empty vectors or MYB gene recombinant plasmids were cultured overnight in SD/-T medium to OD = 1, then diluted to OD600 = 0.4, 0.04, 0.004, and 0.0004. A total of 10 μL of each sample was spotted onto the appropriate deficient media. After growing at 30 °C for 5 days, images were taken. The names of the transformants are listed on the left side of the image.

The transcriptional activity of the identified MYB genes was assessed using a yeast-based system. The experimental setup included pGBKT7 as a negative control, pGBKT7-Lam as a repressor control, and pGBKT7-VP16 as an activator control. As shown in Figure 6, yeast transformants carrying pGBKT7-Os01g0192300, pGBKT7-Os06g0637500, and pGBKT7-Os06g0669700 demonstrated normal growth on SD/-Ade/-His selective media but failed to grow on SD/-Ade/-His/-Trp media. Transformants with pGBKT7-VP16-Os01g0192300, pGBKT7-VP16-Os06g0637500, and pGBKT7-VP16-Os06g0669700 showed no repression, indicating that these genes possess transcriptional activation activity, albeit weaker than the positive control. Conversely, transformants containing pGBKT7-VP16-Os02g0685200 and pGBKT7-VP16-Os09g0106700 failed to grow on SD/-Ade/-His/-Trp media, demonstrating transcriptional repression activity similar to the negative control pGBKT7-VP16-Lam. Os05g0579600 showed no clear transcriptional activation or repression, warranting further investigation through in vivo plant experiments.

### 2.8. Expression Profile of Key Heat-Response MYB Genes Under Other Abiotic Stresses

**Figure 7:**
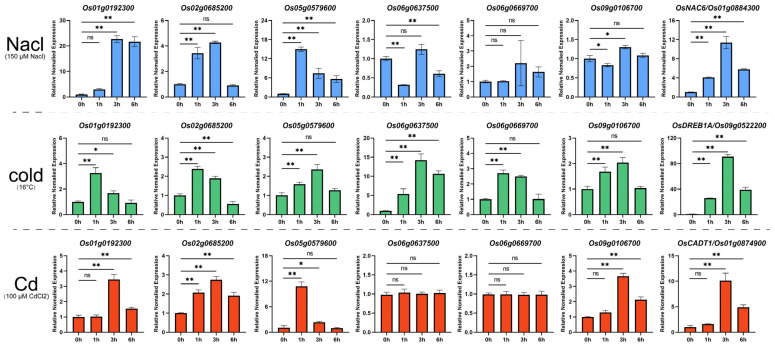
Expression profiles of OsMYBs under various abiotic stresses. This figure presents the RT-qPCR analysis results of six MYB transcription factors and marker genes under different stress conditions. The bar graph illustrates the expression changes of each gene before and after treatment, with the bar height representing relative expression levels. The asterisks indicate statistically significant differences (* for *p*-value < 0.05, ** for *p*-value < 0.01), and ns indicates no significant difference. *OsNAC6*, *OsDREB1A*, and *OsCADT1* were used as marker genes for salt, cold, and cadmium stress, respectively.

Given that certain transcription factors are involved in multiple abiotic stress responses [25,26,27,28], we examined the transcriptional responses of these six genes under salt, cold, and heavy metal (cadmium) stress conditions using RT-qPCR (Figure 7). Under salt stress, *Os02g0685200* and *Os05g0579600* showed rapid upregulation (within 1 h) in rice roots, while *Os01g0192300* exhibited a 21-fold increase after 3 h, maintaining this upregulation for up to 6 h. Most genes responded to cold stress (16 °C), with *Os06g0637500* reaching its maximum transcription level (15-fold increase) after 3 h. Other genes showed a more moderate responses to cold stress, with upregulation below 4-fold. *Os05g0579600* demonstrated high sensitivity to cadmium stress, showing a 10-fold expression increase after 1 h. *Os01g0192300*, *Os02g0685200*, and *Os09g0106700* showed approximately a 4-fold upregulation following cadmium treatment, while *Os06g0637500* and *Os06g0669700* remained unchanged. These results demonstrate the diverse stress response patterns of the identified OsMYB genes, suggesting their potential roles in multiple stress response pathways.

## 3. Discussion

Heat stress in rice activates a multilayered regulatory network governed by several transcription factor families. Classic HSFs rapidly upregulate heat-shock proteins, whereas NAC, WRKY, and AP2/EREBP TFs extend the response to other stress dimensions [17,20,34,35,36]. Within this framework, the MYB family has emerged as a critical node. MYBs can either activate or repress downstream genes, thereby tuning stress pathways in concert with HSFs and related regulators [27,30].

Utilizing transcriptome data to identify novel genes has become a prevalent and highly effective strategy. In transcriptomic analyses of heat stress across various species, the MYB transcription factor family exhibited significant alterations post-treatment [37,38,39,40,41]. In this study, we identified 134 MYB family transcription factors that exhibited significant responsiveness to high-temperature stress during the rice seedling stage by RNA-seq analysis. This indicates that the MYB family may play a significant role in the early thermal response of rice. In particular, six MYB genes (*Os01g0192300*, *Os02g0685200*, *Os05g0579600*, *Os06g0669700, Os06g0637500*, and *Os09g0106700*) previously associated with non-thermal stress pathways emerged as novel heat-responsive regulators, displaying pronounced transcriptional dynamics under a thermal challenge [42,43,44]. Our subcellular localization showed that all six of these genes encode nuclear-localized proteins; the nuclear localization of MYBs supports their role in direct transcriptional control [45,46,47,48,49]. Notably, MYB factors often serve as nodes of cross-talk, integrating heat stress signals with hormone and secondary metabolism pathways to optimize plant survival [27,29]. Therefore, the present study, in addition to observing the changes in the expression of these genes under heat stress, also examined their hormonal as well as other abiotic stresses in response. Below, we discuss each gene’s expression profile and characteristics, and infer its potential role in the heat stress regulatory network in light of known stress regulators.

*Os01g0192300* displayed a slightly slower response to heat, remaining elevated for a long duration and with transcript levels peaking by ~2 h into stress conditions. The protein encoded by *Os01g0192300* also behaves as a transcriptional activator in our assays, localizing to the nucleus. In addition to heat, *Os01g0192300* was modestly induced by high salinity. As mentioned above, numerous MYB TFs function in cross-tolerance. For instance, *ONAC023* is a central regulatory factor in rice that controls drought tolerance and heat tolerance [35]. It positively regulates the drought and heat tolerance of rice at both the vegetative and reproductive stages [35]. Another NAC TF, *OsNAC6* (*SNAC2*), is induced by cold, salt, drought, ABA, JA, mechanical injury, and infection by the rice blast fungus [50,51]. By analogy, *Os01g0192300* possibly regulates downstream genes that confer generalized stress tolerance (such as osmoprotectant biosynthesis or membrane-stabilizing proteins).

*Os02g0685200* was one of the most rapidly upregulated genes under heat stress in our dataset. Transcript levels rose within 30 min of stress exposure and then tapered off at later time points. The swift, robust activation of *Os02g0685200* parallels the behavior of known early heat-response regulators like HSFs in rice [34], implying that *Os02g0685200* may occupy an upstream position in the regulatory network. Consistent with previous studies, the *Os02g0685200* protein showed clear nuclear localization [42]. Although *Os02g0685200* did not exhibit transcriptional activation in our yeast system, its demonstrated transactivation activity in rice protoplasts [42] leads us to hypothesize that this functional discrepancy may arise from fundamental differences between heterologous yeast systems and plant-specific cellular contexts, particularly regarding post-translational modification machinery or species-specific cofactors required for MYB protein activation.

Compared with early heat-responsive MYB genes (like *Os02g0685200*), *Os06g0637500* accumulates more slowly, peaking at ~2 h after heat exposure and ~3 h after cold treatment, yet it responds rapidly to exogenous ABA (within 1 h) and more slowly to auxin (≈3 h). Bioinformatics analyses reveal that its promoter is densely populated with hormone- and stress-related motifs, including 5 ABREs, a single auxin-responsive TGA element, and paired TGACG/CGTCA boxes linked to jasmonate signaling, among others. The encoded protein localizes to the nucleus and functions as a transcriptional activator. Prior work showed that OsMYB102 delays senescence by enhancing ABA catabolism and that the heterologous expression of *OsMYB102* in Arabidopsis likewise postpones leaf senescence, albeit with increased sensitivity to salt and drought stress [52,53]. Comparative genomic surveys further indicate that rice MYB genes containing both ABRE and TGACG/CGTCA motifs are often expressed under multiple abiotic stresses [44]. These observations lead us to propose that *Os06g0637500* functions during the maintenance phase of heat (and cold) acclimation: once ABA levels rise, it becomes active and, together with other ABA-responsive partners, reinforces the expression of late-acting protective genes.

The promoter of *Os06g0669700* is enriched with cis-acting elements, including a low-temperature-responsive element (LTR) and multiple ABRE motifs, and the gene is strongly induced by abscisic acid (ABA). This agrees with the bidirectional induction we observed under both heat and cold stress, suggesting that *Os06g0669700* may be activated by upstream factors in a temperature-sensing pathway. Given its sustained heat inducibility, pronounced activation activity, and nuclear localization, similar to *Os06g0637500*, we propose that *Os06g0669700* may work in concert with ABA-responsive bZIP/ABF and DREB-type regulators to bolster the expression of cellular homeostasis-related genes during extended heat acclimation [54,55], although direct MYB–bZIP/DREB interactions under heat stress remain to be elucidated.

*Os09g0106700* is markedly upregulated by both ABA and jasmonic acid (JA), and its promoter harbors four ABREs and two CGTCA motifs, which are fully consistent with this dual hormone responsiveness. Coupled with its concurrent induction by heat and cadmium stress, this “ABA/JA–ROS” resonance pattern implies that *Os09g0106700* operates at the convergence point of heat and oxidative stress signaling. Comparable multi-stress, multi-hormone integration has been documented for *AtWRKY39* in Arabidopsis and *ZmWRKY106* in maize [56,57]. Thus, *Os09g0106700* may act as a critical amplifier of coordinated thermotolerance and oxidative stress resistance.

Particularly noteworthy is *Os05g0579600*, which exhibits unique subcellular localization characteristics, being present in both the nucleus and the cytoplasm. This pattern resembles *OsNTL3*, a NAC transcription factor known to translocate from the plasma membrane to the nucleus under heat stress [12]. The ntl mutant, lacking a functional domain, shows heat stress sensitivity, while the truncated form of *OsNTL3* (lacking a transmembrane domain) significantly enhances heat tolerance in rice seedlings when induced. This underscores the crucial role of transcription factor subcellular localization in their functionality. Interestingly, *Os05g0579600* showed neither transcriptional activation nor repression in yeast, despite containing a MYB-like domain, suggesting potential non-canonical transcription factor functions. Further investigation into *Os05g0579600*’s roles in different subcellular compartments and its potential membrane-to-nucleus translocation mechanism will provide new insights into rice heat tolerance mechanisms.

While this study elucidates potential roles of several MYB genes in the heat stress response, numerous questions remain for future investigation. Key areas include determining whether gene responses to various stress conditions are mediated through synergistic hormone regulation, and validating the potential membrane-to-nucleus translocation of *Os05g0579600*. Due to limitations of the experimental system, the transcriptional activities of these transcription factors and the identification of their downstream targets require further investigation and validation in rice-specific materials. Although some limitations remain, our findings establish a valuable basis for dissecting the intricate regulatory roles of MYB TFs in response to heat stress. Continued efforts will be directed towards functional validation in rice to clarify their contributions to thermotolerance mechanisms.

## 4. Materials and Methods

### 4.1. Screening and Identification of OsMYB Genes in Rice

The genome of rice (*Oryza sativa* subsp. *japonica*, version: IRGSP-1.0) and corresponding proteome file were obtained from Ensembl Plants (https://plants.ensembl.org/, accessed on 18 March 2025). Protein sequences containing MYB DNA-binding domains in Arabidopsis were obtained from the TAIR database (https://www.arabidopsis.org/, accessed on 18 March 2025). The MYB domain seed sequences were downloaded from InterPro database (https://www.ebi.ac.uk/interpro/entry/pfam/PF00249/entry_alignments/?type=seed, accessed on 16 May 2025). BLASTp (v2.15.0+) was performed using the MYB domain seed sequence with the rice proteome file, and the E-value threshold was set to 0.01. The BLASTp results were filtered by hmmsearch (v3.3.2), only the protein sequences containing the MYB domain (PF00249) were retained, and the E-value threshold was set to 0.001 [58,59]. Nucleotide length, predicted protein length, molecular weight, and isoelectric point were obtained from the Rice Genome Annotation Project (https://rice.uga.edu/index.shtml, accessed on 18 March 2025).

### 4.2. Phylogenetic Tree Analysis

To investigate the evolutionary relationships between OsMYB genes in various plant species, the MYB protein sequences of *A. thaliana* and *O. sativa* were used to construct an unrooted phylogenetic tree. Multiple-sequence alignment was performed using Clustal Omega (v1.2.4) software, and the tree was constructed according to the neighbor-joining (NJ) method with the p-distance substitution model in MEGA (v11) software. We used 1000 replicates in a bootstrap analysis to determine a support value for each branch. Phylogenetic tree visualization was performed by itol (https://itol.embl.de/, accessed on 18 March 2025) and sequence classification was based on the identification results of hmmsearch.

### 4.3. Plant Materials, Growth Conditions, and Sample Treatment

Two-week-old rice seedlings (Zhonghua 11) were exposed to different types of abiotic stress. The seedlings were placed in a plant incubator for heat treatment (45 °C, 80% humidity), with samples collected from the aerial parts before heat treatment (designated as 0 h) and at 30 min, 1 h, and 2 h post-treatment. The seedlings were then transferred to a cold room (16 °C, 45% humidity) to simulate cold stress. A 15% PEG 6000 solution and a 100 µM CdCl_2_ solution were used to simulate drought and heavy metal stress, respectively. These three treatments were sampled at 0 h, 1 h, 3 h, and 6 h, with aerial parts collected for cold and drought treatments and roots for cadmium stress.

### 4.4. RNA Isolation, RNA-Seq and Quantitative Real-Time PCR

RNA was extracted from rice tissues under different treatments using the Trizol extraction method. Library preparation and sequencing were performed by Geneplus Biotechnology Co., Ltd. (Beijing, China). The raw data were filtered using Fastp (v0.20.0) software. Ribosomal RNA was removed using Bowtie2 (v2.3.5.1), and the clean reads after ribosomal RNA removal were aligned to the reference genome using STAR (v2.7.6a). Transcript quantification was performed using StringTie (v2.0.4). After obtaining the gene quantification results, differential expression analysis was conducted using the DESeq2 package (v1.26.0) in R, with significantly different genes selected based on the criteria |log2FC| > 1 and *p*-value < 0.05. The pheatmap package (v1.0.12) in R was used to visualize differential gene expression via heatmaps.

For heat, cold, drought, and cadmium treatments, RNA was reverse transcribed to synthesize cDNA, followed by quantitative real-time PCR. The fluorescent quantitative primers are provided in the Supplementary Table. Relative quantification was calculated using the 2^^−▲▲CT^ method, with the 0 h time point of each treatment serving as the control. All primer sequences used are listed in Table S4. The rice Actin gene (*Os03g0718100*) was used as an internal standard [4,60].

### 4.5. Analysis of Cis-Elements in OsMYB Promoter Regions

The coordinates of the target genes were retrieved from the reference genome annotation file. Using Bedtools (v2.31.1), the 2000 bp sequence upstream of the target genes’ start codon was extracted from the reference genome. The prediction of *cis*-elements was performed using the online tool PlantCARE (https://bioinformatics.psb.ugent.be/webtools/plantcare/html/, accessed on 18 March 2025). The prediction results were visualized using the GSDS2.0 online tool (https://gsds.gao-lab.org/, accessed on 18 March 2025) [61,62,63].

### 4.6. Hormone Expression Level Analysis

Gene expression data under different hormone treatments at various time points for six candidate genes were retrieved from the RicExPro website (https://ricexpro.dna.affrc.go.jp/GGEP/, accessed on 18 March 2025). After obtaining the data, gene expression levels were visualized using the R package pheatmap (v1.0.12).The expression data derived from the database come from the dual-color microarray hybridization system. Cy5 (cyanine 5) and Cy3 (cyanine 3) represent two different colors of fluorescence signals. This database determines the expression level of genes by comparing the ratios of these two [64]. 

### 4.7. Subcellular Localization Assays

To investigate the subcellular localization of the OsMYB protein, the coding region of *Os01g0192300* was inserted between the SacI/BamHIrestriction sites of the p1300-35s-eGFP vector to construct the recombinant vector p1300-35s-*Os01g0192300*-GFP (other genes were constructed in the same manner). The cloning procedure adhered to the guidelines provided in the NovoRec^®^ One-Step PCR Cloning Kit (Novoprotein, Beijing, China). For the control, an empty GFP vector and NLM-mCherry (a nuclear localization marker, purchased from Coolaber, Beijing, China) were co-transfected into one-month-old tobacco leaves. The experimental group consisted of MYB-GFP and NLM-mCherry co-transfected into tobacco leaves. After Agrobacterium infiltration, tobacco plants were incubated at 25 °C for 48 h. Fluorescence signals were observed using a Zeiss LSM 880NLO confocal microscope (Carl Zeiss, Oberkochen, Germany). The primer sequences used in this experiment are provided in the appendix. All primer sequences used are listed in Table S1.

### 4.8. Yeast Transcriptional Activity Assays

Herpes simplex virus protein 16 (VP16) is a transcriptional activator that can induce downstream gene expression. The VP16 activation domain is fused with GAL4 BD and expressed in the pGBKT7-VP16 (BD-VP16) vector, which is then transformed into Y2HGold cells. The transcriptional activation by VP16 activates the expression of the yeast reporter genes HIS3 and ADE2, allowing the yeast to grow normally on histidine and adenine-deficient media. The transcription factor gene is fused with BD or BD-VP16, and if transcription factor X activates downstream gene expression, it will induce the expression of the downstream gene. Compared to the control strain containing BD, the strain containing BD-X will grow on the deficient medium. If transcription factor X inhibits the transcription of downstream genes, the strain containing BD-VP16-X will show inhibited growth on the deficient medium compared to the control strain containing BD-VP16 (pGBKT7-VP16-Lam as the repressor control). Yeast transformation followed standard protocols. Several yeast transformants were selected and verified using the primers listed in the appendix for positive clone identification. The positive yeast strains were cultured in SD/-Trp liquid medium and adjusted to an OD600 of 0.4. Then, they were serially diluted by 10, 100, and 1000 times (i.e., OD600 = 0.4, 0.04, 0.004, 0.0004). A total of 10 μL of each diluted yeast solution was spotted onto SD/-Trp, SD/-His/-Trp, and SD/-Ade/-His/-Trp plates. The plates were incubated at 28–30 °C for 2–3 days, and the growth of each sample on the different selective plates was observed to determine whether the target protein has transcriptional activation or inhibition activity. All primer sequences used are listed in Table S1.

## Figures and Tables

**Table 1:** Physicochemical properties of selected OsMYB genes.

RAP Locus	MSU Locus	Chr *	CDS Coordinates (5′-3′)	Nucleotide Length (bp)	Number of Amino Acids (aa)	Predicted Molecular Weight (Mw/Da)	Predicted pI
*Os01g0192300*	*LOC_Os01g09640*	1	4,973,555–4,971,171	933	311	33,037.4297	9.97
*Os02g0685200*	*LOC_Os02g46030*	2	28,044,149–28,041,115	1476	492	51,674.4492	6.51
*Os05g0579600*	*LOC_Os05g50340*	5	28,846,050–28,845,445	273	91	10,371.8096	9.42
*Os06g0637500*	*LOC_Os06g43090*	6	25,900,817–25,899,442	936	312	33,377.3398	8.10
*Os06g0669700*	*LOC_Os06g45840*	6	27,740,731–27,734,722	1032	344	36,246.0391	7.17
*Os09g0106700*	*LOC_Os09g01960*	9	658,374–656,425	960	320	34,573.3008	10.21

RAP Locus: Gene ID based on the Rice Annotation Project database. MSU Locus: Gene ID from the Michigan State University rice genome annotation (RGAP).CDS coordinates: Genomic start and end positions of the coding DNA sequence (CDS). pI: Theoretical isoelectric point of the encoded protein.* The chromosome on which the gene is located.

## Data Availability

The data supporting the reported results are provided in the Supplementary Materials attached to this manuscript.

## Supplementary Materials

The following supporting information can be downloaded at https://www.mdpi.com/article/10.3390/plants14121784/s1. Figure S1: Phylogenetic analysis of MYB transcription factors in *O. sativa* and *Arabidopsis thaliana*. The phylogenetic tree was constructed based on the MYB protein sequences from *O. sativa* and *A. thaliana*. The proteins are grouped into five distinct clusters (Group 1–Group 5), highlighted with different colors (green, pink, yellow, orange, and blue, respectively). Table S1: Sequences of primers used for RT-qPCR and gene cloning. Table S2: MYB protein sequences in *O. sativa* and *A. thaliana*. Table S3: Expression level of MYB genes during 2 h heat treatment.
